# Segmentation of Coronary Angiograms Using Gabor Filters and Boltzmann Univariate Marginal Distribution Algorithm

**DOI:** 10.1155/2016/2420962

**Published:** 2016-09-25

**Authors:** Fernando Cervantes-Sanchez, Ivan Cruz-Aceves, Arturo Hernandez-Aguirre, Juan Gabriel Aviña-Cervantes, Sergio Solorio-Meza, Manuel Ornelas-Rodriguez, Miguel Torres-Cisneros

**Affiliations:** ^1^Centro de Investigación en Matemáticas (CIMAT), A.C., Jalisco S/N, Col. Valenciana, 36000 Guanajuato, GTO, Mexico; ^2^CONACYT, Centro de Investigación en Matemáticas (CIMAT), A.C., Jalisco S/N, Col. Valenciana, 36000 Guanajuato, GTO, Mexico; ^3^DICIS, Universidad de Guanajuato, Comunidad de Palo Blanco s/n, 36885 Salamanca, GTO, Mexico; ^4^Unidad de Investigación, UMAE 1 Bajío, IMSS, León, GTO, Mexico; ^5^Tecnológico Nacional de México-Instituto Tecnólogico de León, Av. Tecnológico s/n, Fracc. Ind. Julián de Obregón, 37290 León, GTO, Mexico

## Abstract

This paper presents a novel method for improving the training step of the single-scale Gabor filters by using the Boltzmann univariate marginal distribution algorithm (BUMDA) in X-ray angiograms. Since the single-scale Gabor filters (SSG) are governed by three parameters, the optimal selection of the SSG parameters is highly desirable in order to maximize the detection performance of coronary arteries while reducing the computational time. To obtain the best set of parameters for the SSG, the area (*A*_*z*_) under the receiver operating characteristic curve is used as fitness function. Moreover, to classify vessel and nonvessel pixels from the Gabor filter response, the interclass variance thresholding method has been adopted. The experimental results using the proposed method obtained the highest detection rate with *A*_*z*_ = 0.9502 over a training set of 40 images and *A*_*z*_ = 0.9583 with a test set of 40 images. In addition, the experimental results of vessel segmentation provided an accuracy of 0.944 with the test set of angiograms.

## 1. Introduction

The automatic segmentation of coronary arteries in X-ray angiograms represents an important and challenging task for systems that perform computer-aided diagnosis, as it can help specialists in diagnosing and monitoring vascular abnormalities. The two key challenges in X-ray angiograms are the nonuniform illumination and low contrast between coronary arteries and image background. Due to these disadvantages, the blood vessel segmentation problem has been commonly addressed in two different steps: detection also known as enhancement and classification of vessels. The first step is performed for emphasizing vessel-like structures while reducing image noise, and the second step focuses on applying a classification technique to segment vessel pixels from the image background.

In literature, several methods have been introduced for the automatic detection and segmentation of blood vessels for different type of clinical studies. Most of the reported methods are based on mathematical morphology [1–4], Gaussian matched filters [5–9], Hessian-based methods [10–12], and Gabor filters [13], which have been used for different clinical studies such as angiography images of the brain [14] and retinal fundus images [15, 16]. Generally, these methods require an optimization process to select the most suitable values for each specific application. The morphology-based method of Eiho and Qian [1] only depends on the size *S* of the structuring element of the single-scale top-hat operator to detect vessel-like structures. The Gaussian matched filters (GMF) [5] have four parameters to be tuned: the parameter *L* that represents the length of the vessel segment to be detected, *σ* that represents the spread of the intensity profile, *T* which is the position where the Gaussian curve trails will cut, and *κ* that defines the number of evenly spaced filters of the filter bank. The method of Kang et al. [8, 9] uses the four GMF parameters (*L*, *σ*, *T*, *κ*) and the scale parameter (*S*) of the single-scale top-hat operator to perform the vessel detection task. The method of Wang et al. [12] depends on the range to define the spread of the Gaussian curve (*σ*) and the corresponding step size (*δ*). The most common strategy to determine the optimal value for each parameter is based on an exhaustive global search over a training set of images. In this global search, the area under the receiver operating characteristic (ROC) curve is computed, and the parameters with the highest area are chosen as the optimal values. The main disadvantage of this strategy is the high computational time.

Moreover, the single-scale Gabor filters designed by Rangayyan et al. [15, 16] are governed by two discrete parameters (*κ*, *τ*) and one continuous parameter (*l*). These parameters are used to generate the Gabor kernel in order to be convolved with the input image in the frequency domain. The parameter *κ* represents the number of evenly spaced filters in the range [−*π*/2, *π*/2], the parameter *τ* is the average thickness of the vessels, and the parameter *l* represents the elongation of the Gabor kernel. To determine the optimal values for the Gabor filter parameters, Rangayyan et al. [16] used the exhaustive global search over a predefined range of values for each parameter. In general, the Gabor filters present superior performance for detecting vessel with low contrast; however, due to the fact that these filters are performed in the frequency domain, their training stage is computationally more expensive than the spatial methods discussed above.

To avoid an exhaustive global search and solve the parameter optimization problem, population-based methods such as Estimation of Distribution Algorithms (EDAs) [17] can be introduced. EDAs are stochastic methods that incorporate statistical knowledge of potential solutions to solve discrete or continuous optimization problems. In the present work, we propose the use of the Boltzmann univariate marginal distribution algorithm (BUMDA) [18] from the family of EDAs for selecting the most suitable parameters of the single-scale Gabor filters while reducing the computational time with respect to an exhaustive global search. This proposed method is applied for detecting coronary arteries in X-ray angiograms, where the optimization process is carried out over the three Gabor filter parameters (*κ*, *τ*, *l*). The performance of the proposed method is compared with those obtained using five state-of-the-art vessel detection methods by computing the area under the ROC curve.

The remainder of this paper is organized as follows. In Section 2, the fundamentals of the single-scale Gabor filters, Boltzmann univariate marginal distribution algorithm, and the parameter optimization process are described in detail. The experimental results are discussed in Section 3, and conclusions are given in Section 4.

## 2. Methods

In general, the contrast between vessel-like structures and background pixels in X-ray angiograms is low; consequently, the single-scale Gabor filter and BUMDA technique for improving its performance are of interest in the present work; these methods are described and analyzed in the present section.

### 2.1. Single-Scale Gabor Filter

The Gabor filter represents a Gaussian function modulated by a sinusoid [13]. Since this filter can be rotated to different angles by applying a geometric transformation, it is useful to detect tubular structures at different orientations through a directional filter bank [19]. The main kernel of a Gabor filter can be defined as follows:

(1) gx,y=12πσxσyexp⁡−12x2σx2+y2σy2cos⁡2πfox,

where *σ*_*x*_ and *σ*_*y*_ represent the standard deviation values of the Gaussian function and *f*_*o*_ is the frequency of the modulating sinusoid. In the single-scale Gabor filter designed by Rangayyan et al. [15, 16], three parameters to control the Gabor filter response were introduced. The first parameter $\tau =\sigma_{x}2\sqrt{2\ln2}$ represents the average thickness (in pixels) of the vessel-like structures to be detected. The second parameter *l* is used to control the length of the kernel as *σ*_*y*_ = *lσ*_*x*_, and the third parameter *κ* is used to rotate the Gabor kernel at different angular resolutions (*θ*) in the range [−*π*/2, *π*/2], obtaining *κ* = 180/*θ* oriented filters. To acquire the filter response, these kernels are convolved with the input image, and for each pixel, the maximum response over all orientations is conserved.

**Figure 1:**
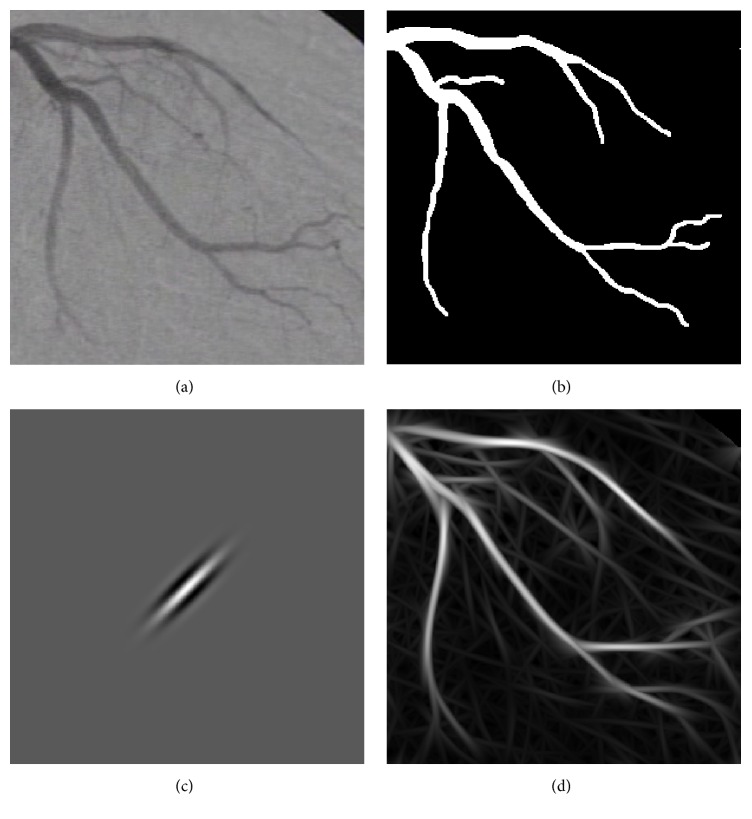
(a) Original X-ray coronary angiogram. (b) Ground-truth of angiogram in (a). (c) Gabor filter kernel with *l* = 2.9, *τ* = 15, and *θ* = 45°. (d) Resulting enhanced image using the angiogram in (a) and the filter bank of Gabor kernels with the parameters {*l*, *τ*} of (c), with *κ* = 180.

To obtain the best performance of the single-scale Gabor filters, the discrete parameters *τ*, *κ* and the continuous parameter *l* have to be determined. In Figure 1, an X-ray coronary angiogram along with its hand-labeled image (ground-truth) as drawn by a specialist are presented.  Figure 1(c) illustrates a Gabor kernel with values *l* = 2.9, *τ* = 15, and *θ* = 45°, and the enhancement result with a filter bank using *κ* = 180 is given in Figure 1(d).

### 2.2. Boltzmann Univariate Marginal Distribution Algorithm

The Boltzmann univariate marginal distribution algorithm (BUMDA) [18] represents a population-based method from the family of Estimation of Distribution Algorithms (EDAs) used to solve numerical optimization problems in discrete or continuous domain. BUMDA is based on a Normal-Gaussian model to approximate the Boltzmann distribution, which is used to generate new potential solutions at each generation *t*. In the selection process, a threshold value *φ*_*t*_ is computed in order to discriminate the individuals according to the fitness value *g*(*x*). The individuals with the best performance are used to form the selection set *S*. In the general case, for each generation, the mean vector of the Gaussian model can be estimated as follows:

(2) $$\begin{array}{c} \mu_{t}=\frac{\sum_{i=1}^{N_{S}}\bar{g}\left(x_{i}\right)x_{i}}{\sum_{i=1}^{N_{S}}\bar{g}\left(x_{i}\right)}, \end{array}$$

where vector *x*_*i*_ is the set of attributes of individual *i*, *N*_*S*_ represents the number of individuals in *S*, and $\bar{g}(x_{i})$ is the difference between the current individual and the worst individual in *S*. In further iterations, the threshold value *φ* forces the mean estimation of the Gaussian model to converge to the optimal solution.

Moreover, as convergence criterion, the variance estimation can be computed as follows:

(3) $$\begin{array}{c} v_{t}=\frac{\sum_{i=1}^{N_{S}}\bar{g}\left(x_{i}\right){\left(x_{i}-\mu_{t}\right)}^{2}}{1+\sum_{i=1}^{N_{S}}\bar{g}\left(x_{i}\right)}. \end{array}$$

This convergence criterion tends to zero by evaluating the variance between *x*_*i*_ and *μ*_*t*_. According to previous description, BUMDA can be implemented by the following procedure (http://www.cimat.mx/~ivvan/public/bumda.html):

(1) Establish minimum variance *v*_min_ as convergence criterion.
(2) Generate *N individuals* with attributes randomly selected.
(3) Select the best* individuals* with function evaluation above *φ* and from *S*.
(4) Calculate *μ*_*t*_ using (2) from *S*.
(5) Calculate *v*_*t*_ using (3) from *S*.
(6) Generate *N* new* individuals* with attributes sampled from a marginal Gaussian distribution with mean *μ*_*t*_ and variance *v*_*t*_.
(7) Insert the individual with the best fitness to the new population.
(8) If *v*_*t*_⪯*v*_min_ then stop; otherwise, repeat from step (3).

### 2.3. Optimization of Single-Scale Gabor Filters

Due to the design of the single-scale Gabor filters, an optimization process to select the most suitable values for the Gabor filter parameters (*κ*, *τ*, *l*) is required. Commonly, the optimal parameter selection is carried out by a training stage, in which an exhaustive global search is performed over varying sets of parameters as applied to a predefined training set of images. Rangayyan et al. [15] proposed a search space for the elongation and average thickness parameters as *l* = {1.7,2.1,…, 4.1} and *τ* = {7,8, 9}, respectively. Subsequently, Rangayyan et al. [16] extended the range of the variables in order to be applied for detecting blood vessels in retinal fundus images as *l* = {1.3,1.7,2.1,…, 17.7,18.1} and *τ* = {1,2, 3,…, 15,16}, keeping constant the number of oriented filters as *κ* = 180. In the exhaustive global search over the training set, for each combination of parameters (*κ*, *τ*, *l*), the Gabor filter response is evaluated by computing the area (*A*_*z*_) under the receiver operating characteristic (ROC) curve. The set of parameters with the highest *A*_*z*_ value is saved, and then, it is directly applied over the independent test set of images. The main disadvantage of using an exhaustive global search for the training stage of Gabor filters is the fact that it is computationally expensive and also that, by using discrete steps in continuous domain, the search space cannot be explored properly.

To avoid a procedure involving an exhaustive global search, in the present work the Boltzmann univariate marginal distribution algorithm has been adopted to perform the optimization process. The use of BUMDA reduces the number of evaluations and computational time of the training stage, while increasing the vessel detection performance by obtaining the optimal Gabor filter parameters.

**Figure 2:**
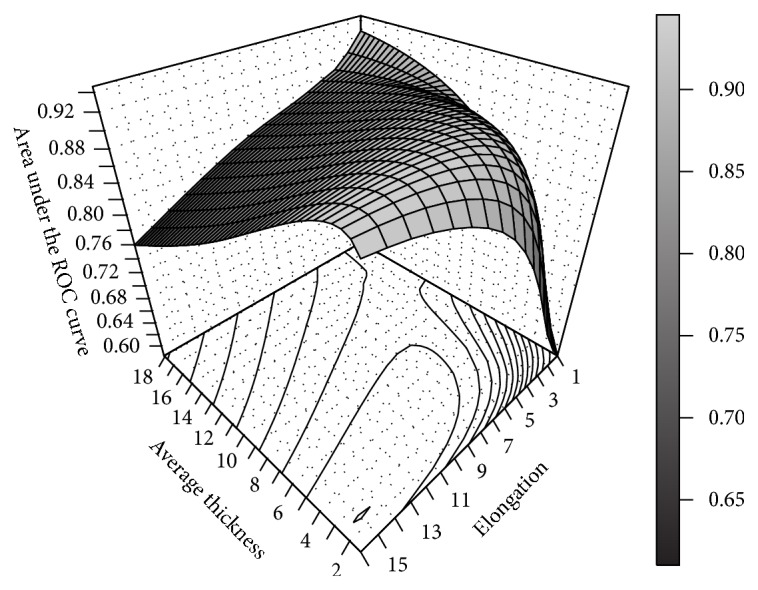
Search space for the Gabor filter parameters (*l*, *τ*) using the area *A*_*z*_ under the ROC curve of the training set of 40 angiograms.

Due to the fact that objective function to be maximized is the area *A*_*z*_ under the ROC curve, the search space of the Gabor filter parameters represented by the elongation (*l*) and the average thickness (*τ*) is illustrated in Figure 2. The range for each variable was defined according to the aforementioned method of Rangayyan et al. [16], since it is appropriate for detecting coronary arteries in X-ray angiograms.

### 2.4. Evaluation Metrics

In order to assess the performance of the vessel detection and segmentation methods, the area under the receiver operating characteristic (ROC) curve and the accuracy measure have been adopted, which are described below.

The ROC curve is a measure to evaluate the performance of a classification system. This curve is calculated by using a sliding threshold to the gray-scale single-scale Gabor filter response in order to plot the true-positive fraction (TPF) against false-positive fraction (FPF), and then, the area *A*_*z*_ under the curve is approximated through the Riemann-sum method.

On the other hand, to evaluate the performance of the vessel segmentation results, the accuracy measure [20] has been applied. The accuracy is the most commonly used metric to evaluate the performance of binary classifiers, and it is defined as the fraction of correctly classified pixels with respect to the total number of pixels in the image as follows:

(4) $$\begin{array}{c} Accuracy=\frac{TP+TN}{TP+FP+TN+FN}, \end{array}$$

where TP and TN are the fractions of correctly classified vessel and nonvessel pixels, respectively, and FN and FP are the fractions of incorrectly classified vessel and nonvessel pixels, respectively.

In both these evaluation metrics, when the vessels and nonvessel pixels obtained from the computational methods are completely superimposed with the ground-truth image, the obtained result is 1 and is 0 when the regions are completely different.

## 3. Results and Discussion

In this section, the vessel detection and segmentation results obtained from the proposed method are analyzed and discussed in different sections. The proposed single-scale Gabor filter optimized by BUMDA (SSG-BUMDA) was implemented on a computer with an Intel Core i3, 2.13 GHz processor, and 4 GB of RAM using the Matlab software version 2013.

The database used in the present work consists of 80 X-ray coronary angiograms of size 300 × 300 pixels. Each angiogram was hand-labeled by a specialist and ethics approval was provided by the Cardiology Department of the Mexican Social Security Institute UMAE León. This database has been divided into the training and testing sets with 40 angiograms in each one in order to evaluate the vessel detection and segmentation results.

### 3.1. Results of Vessel Detection

As part of the vessel detection stage, the performance of the single-scale Gabor filters is compared with four detection methods of the state of the art. The best set of parameters for the detection methods was acquired by varying the range of the variables over the training set and computing the corresponding *A*_*z*_ value. The range for the single-scale Gabor filter parameters was defined by Rangayyan et al. [16] as *l* = {1.3,1.7,2.1,…, 17.7,18.1}, *τ* = {1,2, 3,…, 15,16}, and *κ* = 180. The range of the parameters for the Wang et al. [12] method was established as *σ* = [1,22] with steps of *δ* = [1,5]. Since the morphological method of Eiho and Qian [1] depends of the size of the disk-shaped structuring element for the top-hat operator, the range for the size parameter was defined as *S* = [1,22] pixels. According to the original work of Chaudhuri et al. [5], the Gaussian matched filters parameters were defined as *l* = 9, *σ* = 2.0, *T* = 13, and *κ* = 12, and finally, taking into account the original work of Kang et al. [8, 9], the Gaussian matched filters parameters were established as *l* = 9, *σ* = 1.5, *T* = 13, and *κ* = 6.

**Figure 3:**
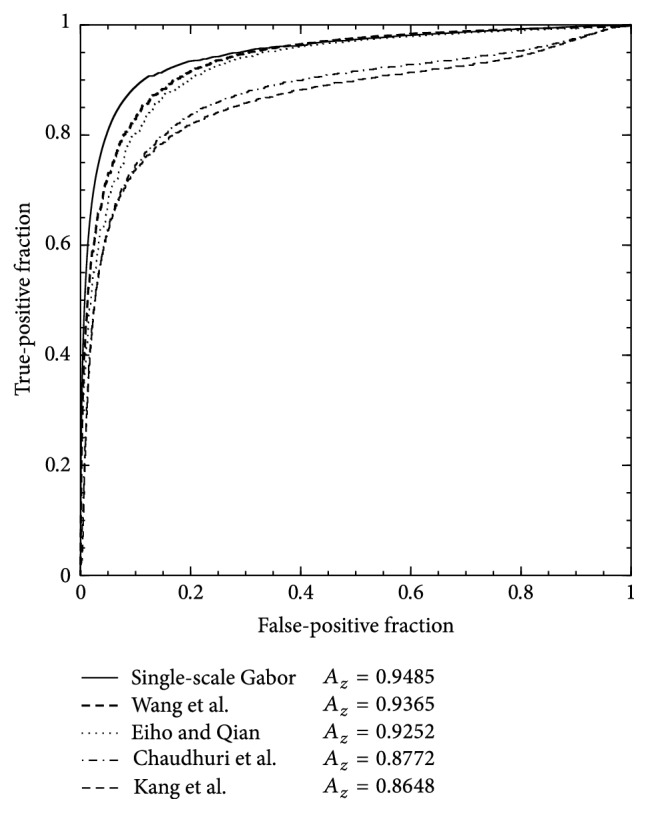
Comparison of ROC curves for vessel detection with the training set, using the single-scale Gabor filters and the comparative methods.

**Figure 4:**
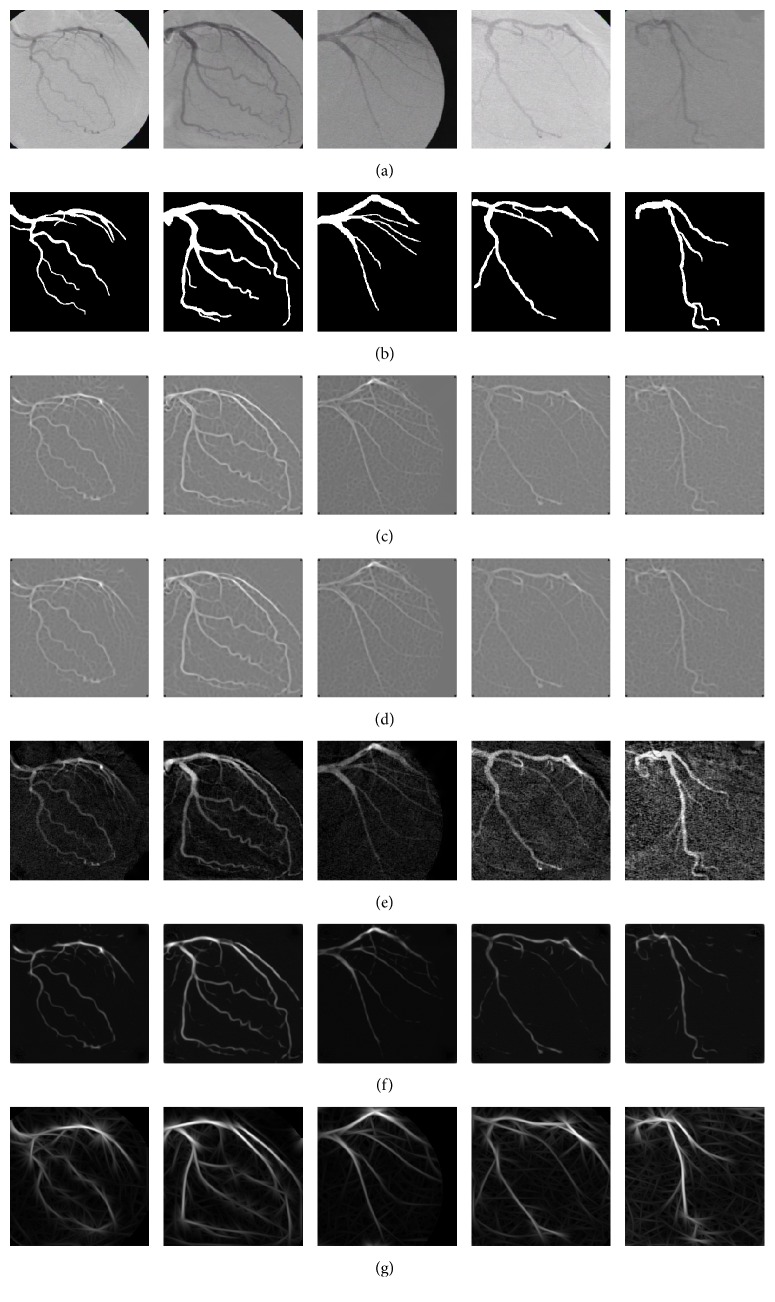
(a) Subset of angiograms from the training set. (b) Ground-truth for the angiograms in (a). (c–g) present the filter response of the methods of Kang et al. [8, 9] (Gaussian filters), Chaudhuri et al. [5], Eiho and Qian [1], Wang et al. [12], and single-scale Gabor, respectively, applied to the angiograms in (a).

In Figure 3, the vessel detection performance of the methods using the training set is presented. The ROC curves are acquired by concatenating the filter responses of the training set to form one large gray-scale image and applying the best set of parameters over the ranges discussed above. This comparative analysis reveals that the single-scale Gabor filters provide the highest performance in vessel detection compared to the comparative methods over the training set of angiograms. In addition, to illustrate the vessel detection results, in Figure 4, a subset of angiograms is introduced along with the filter responses of the comparative methods. By visual inspection, the SSG method presents a higher vessel pixel detection and a better differentiation with the background image than the comparative four methods.

To introduce the vessel detection results obtained from SSG-BUMDA, in Table 1, a statistical analysis is shown. This analysis was performed with 30 runs over the training set of angiograms, where the mean and standard deviation values suggest that BUMDA is robust to perform the optimization task.

On the other hand, since the exhaustive global search and BUMDA can find the optimal solution from the training set, the comparison of searching time or number of evaluations represents one of the advantages of using an evolutionary method. In Table 2, a comparative analysis between the full-search strategy and BUMDA in terms of execution time and number of evaluations to obtain the optimal set of parameters of the single-scale Gabor filters is presented. In this experiment, BUMDA obtains a superior performance compared to the full-search strategy, reducing the computational time. The number of evaluations of BUMDA is computed taking into account the number of individuals (20) and the average number of generations (13.8) over the 30 runs.

From the training set of angiograms, the optimal parameters for each vessel detection method are acquired, and then, the set of parameters are directly applied on the test set. In the method of Eiho and Qian [1], the optimal size for the structuring element of the top-hat operator was determined as *S* = 14 pixels. In the method of Wang et al. [12], the optimal parameters were determined as *σ* = [1,16], with *δ* = 2.5. The optimal parameters for the single-scale Gabor filters using the global search proposed by Rangayyan et al. [16] were determined as *τ* = 15 and *l* = 2.9, and by using BUMDA the optimal parameters were established as *τ* = 15 and *l* = 2.65. In Table 3, the vessel detection results of the comparative methods with the optimal parameters discussed above are illustrated. Since BUMDA performs its optimization strategy over the continuous range of the elongation parameter for the Gabor filters instead of discrete steps, the search space is explored properly. The SSG-BUMDA method obtains the highest *A*_*z*_ value over the test set.

### 3.2. Results of Vessel Segmentation

To classify vessel and nonvessel pixels from the gray-scale Gabor filter responses of the test set, in Table 4, five state-of-the-art automatic thresholding methods have been compared in terms of segmentation accuracy. According to the comparative analysis the interclass variance thresholding method proposed by Otsu [21] has obtained the best segmentation performance; therefore, the interclass variance thresholding method is used for further analysis.

**Figure 5:**
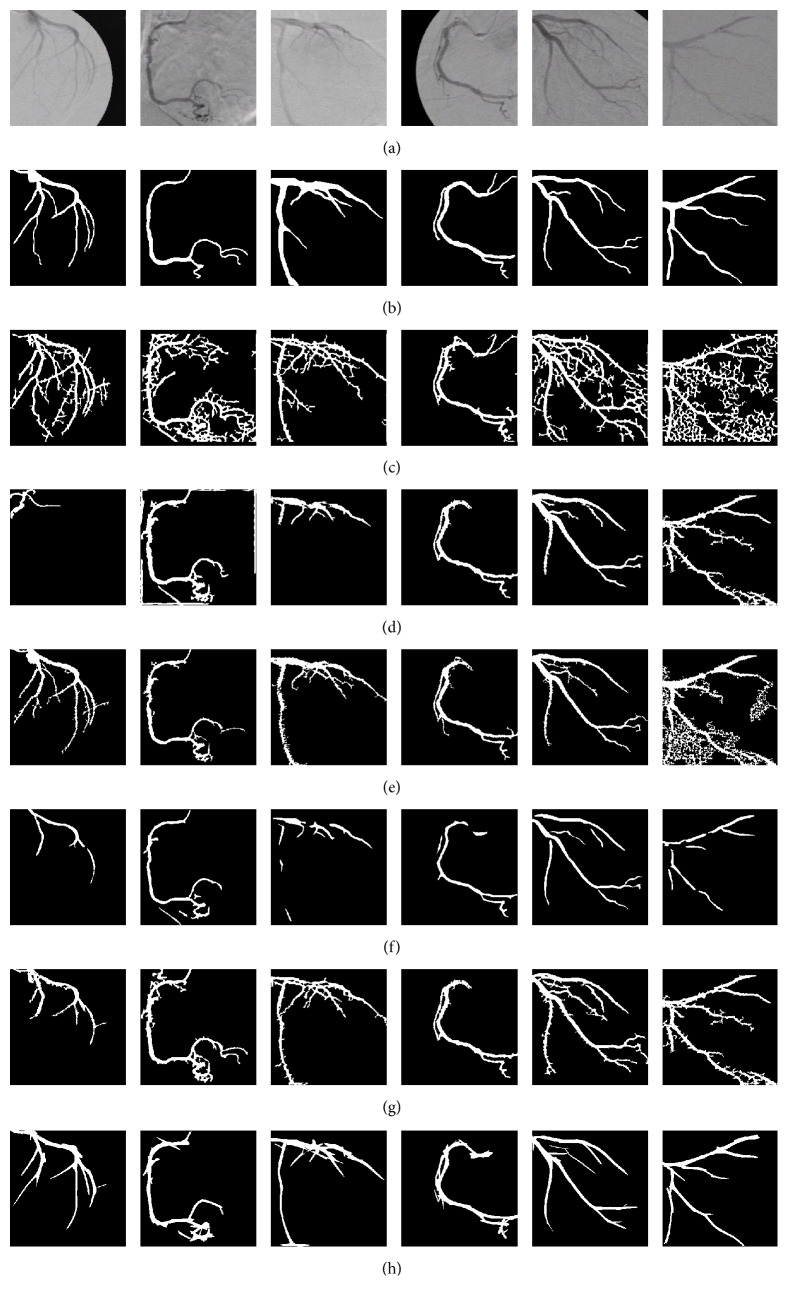
(a) Subset of angiograms from the test set. (b) Ground-truth for the angiograms in (a). (c–h) present the segmentation results of the methods of Chanwimaluang et al. [7], Kang et al. [8], Eiho and Qian [1], Wang et al. [12], Kang et al. [9], and the proposed method, respectively, applied to the images in (a).

Finally, in Table 5, the segmentation results of the proposed method based on single-scale Gabor filters optimized by BUMDA for vessel detection and the interclass variance thresholding method for vessel classification are compared with five state-of-the-art segmentation methods in terms of the accuracy measure. The obtained results show that the proposed method provides the highest vessel segmentation performance in relation to the comparative five specialized vessel segmentation methods with the test set of angiograms. These segmentation results are illustrated in Figure 5, where the proposed method presents an appropriate rate of true-positive pixels and low rate of broken vessels and false-positive pixels.

The vessel detection and segmentation methods of the state of the art provide appropriate performance according to the *A*_*z*_ value and accuracy measure. However, different comparative analyses suggest that the proposed method is robust and suitable for detecting and segmenting vessels in coronary angiograms. These comparative analyses have also shown that the proposed method can be appropriate for systems that perform computer-aided diagnosis in cardiology.

## 4. Conclusion

In this paper, a novel method based on the Boltzmann univariate marginal distribution algorithm for improving the training step of the single-scale Gabor filters has been presented. The statistical results obtained from BUMDA show a superior performance in terms of number of evaluations and computational time compared to a full-search method. The optimization process performed by BUMDA in general achieved a high detection rate of the single-scale Gabor filters taking into account the area under the ROC curve using the training set of angiograms. The best set of parameters determined by BUMDA for the Gabor filters were set as elongation *l* = 2.65 and average thickness *τ* = 15 pixels. The performance of the proposed SSG-BUMDA method has demonstrated to be more efficient compared with five methods of the state of the art achieving *A*_*z*_ = 0.9583 with the test set of angiograms. In the segmentation step, the interclass variance thresholding method has proven to be the most efficient compared with four thresholding methods, obtaining an accuracy rate of 0.944 with the test set of 40 angiograms. According to the experimental results, the proposed method consisting of the application of single-scale Gabor filters optimized by BUMDA for the detection of coronary arteries followed by the interclass variance thresholding method for segmentation can lead to higher accuracy than five state-of-the-art methods for automatic vessel segmentation.

## Figures and Tables

**Table 1:** Statistical analysis using 30 runs of the single-scale Gabor filters with BUMDA over the training set of angiograms.

Method	Maximum	Minimum	Mean	Std. dev.	Median
Proposed SSG-BUMDA	0.9502	0.9394	0.9469	0.0035	0.9483

**Table 2:** Comparison between the single-scale Gabor filters using the exhaustive global search and the proposed method in terms of number of evaluations and execution time.

Method	Evaluations	Execution time (s)
Full-search SSG	(16 × 43) = 688	15521.29
Proposed SSG-BUMDA	(20 × 13.8) = 276	**6755.21**

**Table 3:** Comparison of vessel detection performance with the test set of angiograms, using the proposed and comparative methods.

Method	Area under ROC curve (*A*_*z*_)
Kang et al. [8, 9]	0.9166
Chaudhuri et al. [5]	0.9176
Eiho and Qian [1]	0.9333
Wang et al. [12]	0.9375
Single-scale Gabor [15, 16]	0.9545
SSG-BUMDA	**0.9583**

**Table 4:** Comparative analysis of five thresholding methods of the state of the art using the single-scale Gabor filter response (SSG-BUMDA) over the test set.

Thresholding method	Accuracy
Rosenfeld and De la Torre [22]	0.710
N. R. Pal and S. K. Pal [23]	0.813
Kapur et al. [24]	0.919
Ridler and Calvard [25]	0.923
Otsu [21]	**0.944**

**Table 5:** Comparative analysis of the proposed method (SSG-Bumda/Otsu) with respect to five state-of-the-art segmentation methods using the test set of angiograms.

Segmentation method	Accuracy
Chanwimaluang and Fan [6, 7]	0.852
Kang et al. [8]	0.871
Kang et al. [9]	0.905
Eiho and Qian [1]	0.917
Wang et al. [12]	0.931
SSG-BUMDA/Otsu	**0.944**
